# Participation in genetic screening: testing different outreach methods across a diverse hospital system based patient population

**DOI:** 10.3389/fgene.2023.1272931

**Published:** 2023-10-12

**Authors:** Lindsay Dickey, Ben Gronowski, Kyle Jones, J. B. Rinaldi, Kate Emery, Jon Clemens, Ora Gordon, Keri Vartanian

**Affiliations:** ^1^ Center for Outcomes Research and Education, Portland, OR, United States; ^2^ Center for Clinical Genetics and Genomics for Providence Southern California, Burbank, CA, United States

**Keywords:** genomics, population-level screening, outreach approaches, diverse populations, enrollment

## Abstract

**Introduction:** Genomics has the potential to transform medicine by identifying genetic risk factors that predispose people to certain illnesses. Use of genetic screening is rapidly expanding and shifting towards screening all patients regardless of known risk factors, but research is limited on the success of broad population-level outreach for genetic testing and the effectiveness of different outreach methods across diverse populations. In this study, we tested the effectiveness of Digital Only (emailing and texting) and Brochure Plus Digital (mailed brochure, emailing, and texting) outreach to encourage a diverse patient population to participate in a large hospital system’s whole genome sequencing program.

**Methods:** Disproportionate stratified sampling was used to create a study population more demographically diverse than the eligible population and response rates were analyzed overall and by demographics to understand the effectiveness of different outreach strategies.

**Results:** 7.5% of all eligible patients enrolled in the program. While approximately 70% of patients invited to complete genetic testing identified in their EHR as being Hispanic, Black or African America, Asian, or another non-White race, these patients generally enrolled at lower rates than the overall population. Other underrepresented groups had higher enrollment rates including people with Medicaid coverage (8.7%) and those residing in rural areas (10.6%). We found no significant difference in enrollment rates between our Digital-Only and our Brochure Plus Digital outreach approaches in the overall population, but enrollment rates were significantly higher for Asian patients and patients who resided in rural areas in the Brochure Plus Digital group. Across both outreach approaches, links provided in emails were most commonly used for enrollment.

**Discussion:** Our study reveals expected enrollment rates for proactive outreach by a hospital system for genetic testing in a diverse population. As more hospital systems are adopting population-scale genetic testing, these findings can inform future outreach efforts to recruit patients for genetic testing including those patients traditionally underrepresented in genomics.

## 1 Introduction

Genomics has the potential to transform healthcare by identifying pathogenic genetic variants that predispose otherwise healthy people to disease and informing their risk management and treatment plans (Shen et al., 2022). Genetic testing holds promise to create broadly available personalized care, but today genetic testing is often limited to patients with personal or family history features that suggest hereditary risk, and many patients with these factors are still not identified through routine medical practices. Nearly 2 million Americans are at increased risk of adverse health outcomes due to genetic variants that predispose them to conditions that have been designated by the Centers for Disease Control and Prevention (CDC) as Tier 1 genomics applications (Hereditary Breast and Ovarian Cancer Syndrome (HBOC), Lynch syndrome (LS), and Familial hypercholesterolemia (FH)), named for their significant potential to positively impact public health (Centers for Disease Control and Prevention, 2014). Yet a 2020 study found that over 90% of patients with pathogenic genetic variants predisposing them to a Tier 1 condition had not been previously identified through standard screening practices (Grzymski et al., 2020; Williams, 2022). Proactive population screening may better identify individuals who have increased genetic risk of disease, and these efforts are gaining momentum as federal spending on genomics research increases and programs designed to integrate genomic research into healthcare begin actively enrolling participants or planning to do so in the coming years (All of Us Research Program Investigators, 2019; Tripp and Grueber, 2021; Williams, 2022).

The widespread adoption of genomics will require not just increased funding and integration into health systems, but also intentional outreach strategies to increase potential participants’ knowledge of genomics and buy-in. Engaging populations traditionally underrepresented in genomics, such as people of color, rural patients, and those with lower socioeconomic status, is especially critical to ensure that advances in genetic testing reduce, rather than exacerbate, disparities in health outcomes. Currently, participants of European descent make up a disproportionate number of genome-wide association studies and biobank participants (Bustamante et.al., 2011; Popejoy and Fullerton, 2016). This limits the generalizability of program findings and can have negative impacts on patients from traditionally underrepresented groups who, when they do participate in genetic testing, receive higher rates of ambiguous test results which can lead to anxiety and unnecessary medical interventions (Buchanan et al., 2020; Appelbaum et al., 2022). Khoury et al. (2022) called the equitable implementation of genomics and precision medicine a public health imperative in a 2022 article, as without concerted efforts to engage diverse populations, the benefits of genomics will be unequally distributed and could potentially widen existing health disparities.

The first step in engaging patients in genomic studies is outreach, but existing research is inconclusive about the most effective modes of outreach for proactive population-level screening by hospital systems and the extent to which different communities may have different outreach preferences. Studies often emphasize the importance of relationship building, but individual outreach is not always feasible in population-level genomics interventions given scale. While some health systems have relied on outpatient visits to encourage participation in genomic research (Carey et al., 2016; Lemke et al., 2021), other local and national genetic testing initiatives have blended community engagement, digital and paper-based outreach, and mass media campaigns by utilizing email, social media, TV, radio, and local newspapers (Sperber et al., 2017; All of Us Research Program Investigators, 2019; Hedden et al., 2023). When considering specific outreach strategies to populations traditionally underrepresented in genomics, one study found that active outreach was more effective in leading African American participants to enroll in a genomic clinical trial compared to phone calls (Horowitz et al., 2019), while another found that phone-based recruitment was more effective than mailing materials to increase African American patient participation in genomics (Johnson et al., 2011). Another study recommended a multimodal approach (e.g., email, mailed letters, and phone calls) to reach and engage more diverse study populations in genetic research (Owen-Smith et al., 2020). This lack of consensus points to the need for more research on the effectiveness of different outreach methods to encourage participation in genomic research, particularly for underrepresented populations.

In this study, we used disproportionate stratified sampling to recruit a diverse patient population to participate in a large multi-state hospital system’s Geno4ME (Genomic Medicine for Everyone) program. Geno4ME is a population-level whole genome sequencing (WGS) program that returns a clinical result report for inherited risk related to cancer, cardiovascular disease, and pharmacogenomics. To recruit patients for this program, we tested different combinations of outreach methods, including mail-based paper brochures, emails, and text messages. We examined response rates overall and across demographics to understand the effectiveness of these different outreach strategies for recruitment. This study contributes to our understanding of how health systems can best support population-level recruitment of patients for genomic medicine and consider strategies that are effective across demographics to support more equitable participation and outcomes.

## 2 Methods

### 2.1 Setting

Geno4ME is a whole genome sequencing (WGS) research program implemented by Providence Health & Services (Providence). Once invited to participate, patients are directed to an informational, secure web-based consent platform where they can watch educational videos and determine whether they would like to enroll in the program (all content was offered in English and Spanish). Once consented, patients complete questionnaires and are considered enrolled in the study. At. At-home saliva kits for DNA sample collection are sent to the home address provided by enrollees with prepaid shipping labels to return the kits to a Clinical Laboratory Improvement Amendments (CLIA) and College of American Pathologists (CAP) certified laboratory for DNA extraction, sequencing, and interpretation of variants within genes on the clinical return of results panel. Patients’ results are posted to their individual account in the web-based platform, as well as to their electronic health record (EHR). No cost genetic counseling appointments are scheduled for individuals found to have a mutation associated with increased risk for inherited disease, and pharmacist consultation is provided for individuals with a pharmacogenomic finding that may impact medications they are currently taking. The full study protocol was approved by Providence St. Joseph Health Institutional Review Board (IRB #2020000637).

### 2.2 Sampling

The study population was drawn from patients residing in five states within the Providence system (Oregon, California, Washington, Alaska, and Montana) via Providence EHR. The study eligibility required patients to be 18 years or older and to have had a visit with a primary care provider in the Providence system in the previous 12 months. Eligibility was further restricted to patients who spoke English, Spanish, or American Sign Language as their primary language; who had a viable email address, current address to receive mail, and telephone number; and who had insurance at the time of their most recent visit.

Patient characteristics of race, ethnicity, zip code of current residence, and most recent insurance payer type were extracted from patients’ EHRs and used to generate the study population. Race and ethnicity were combined into a single exclusive variable with race secondary to Hispanic ethnicity. Patients not identified as Hispanic and identified in the EHR data with race of American Indian or Alaska Native, Native Hawaiian or Pacific Islander, and “Two or More Races,” were combined with “Other” race into “Another Race” due to small numbers in the eligible population. Patients without race or ethnicity data (“Patient Refused”, “Unavailable,” or “Unknown”) were collectively labeled “Unknown” for this analysis. Patients with primary payer type other than Medicaid were grouped as “Other Insurance.” Each patient’s most recent residential zip code was designated as urban or rural based on classification in the 2010 Rural Urban Commuting Area Codes table (U.S. Department of Agriculture: Economic Research Service, 2020); zip codes labeled as Metropolitan (codes 1 through 3) were classified as urban, while all less dense areas were classified as rural.

From 750,320 eligible individuals, we used disproportionate stratified sampling to create a population that was more demographically diverse than the eligible population. We sampled six strata, in order: 1) general population (all eligible patients), 2) individuals identifying as Hispanic or Latino/a/x, 3) individuals identifying as Black, 4) individuals identifying as Asian, 5) individuals with Medicaid as primary payer, and 6) individuals residing in a zip code designated as rural. An individual could be eligible for several strata but once selected was not eligible for subsequent draws. For each stratum, individuals were randomly sampled and assigned to one of two outreach methods. The resulting study population contained 20,400 individuals.

### 2.3 Outreach

**FIGURE 1:**
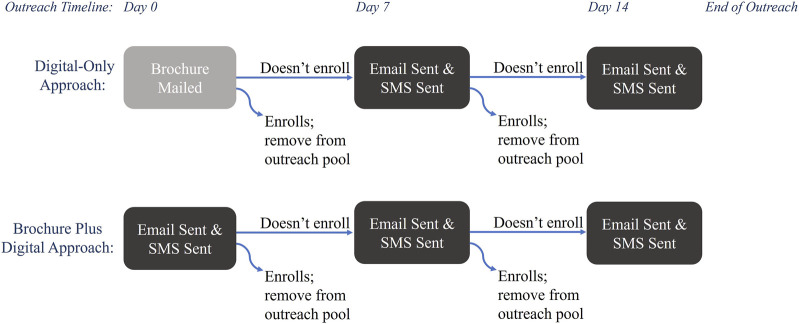
Study outreach workflow.

Outreach was conducted in four waves, from February 2022 to December 2022, and offered in English and Spanish. Each wave contained approximately 5,000 patients divided evenly between two outreach approaches: *Digital-Only* and *Brochure Plus Digital* (Figure 1). The *Digital-Only* outreach approach consisted of contacts via email and text message [also known as short message service (SMS)]; email invitation was followed by SMS within a day and repeated up to two additional times at weekly intervals until patients either enrolled or declined to participate via email or SMS. Individuals in the *Brochure Plus Digital* group received a mailed brochure with information about the study, followed by up to 2 weeks of digital outreach. Digital methods of outreach were sent and tracked within Providence (SMS were delivered through a Providence communications initiative (MPulse/Cadence) and emails were sent via Providence email). The brochures were printed, addressed, and mailed by a third-party organization (Kaye-Smith). Messages sent by email and SMS included a hyperlink and mailed brochures contained a QR code directing patients to a website with information about the study and access to the platform to consent and enroll. Each communication type (email, SMS, and brochure) had a unique link so the path to enrollment could be tracked. Each wave was reviewed weekly to remove patients who enrolled and who declined further outreach. For this analysis, enrollment was open for 3 months after the first invitation was sent, thus enrollment occurred from February 2022 through March 2023. Enrollment data was stored in a secure REDCap database hosted by Providence (Harris et al., 2009).

### 2.4 Analysis

We summarized the demographic distribution of our study population and compared the two outreach population distributions using a Pearson’s χ2 test. We investigated the relationship of outreach approach and enrollment using multiple logistic regression, adjusting for covariates. The covariates included age, sex, race-ethnicity, payer type, and residence type. We used demographic characteristics (race-ethnicity, payer type, and residence type) to divide the population into subgroups, then assessed the strength of the association between outreach type and enrollment with multiple logistic regression, adjusting for the remaining covariates. The associations were reported as incidence rate ratios (IRR) (prevalence of enrollment in the *Brochure Plus Digital* group divided by prevalence of enrollment in the *Digital-Onl*y group) with two-sided 95% confidence intervals (CI). Analyses were conducted using R version 4.2.2 (R Core Team, 2022).

## 3 Results

### 3.1 Participants

20,400 eligible patients were invited to enroll in Geno4ME using the two outreach approaches. The two outreach populations were statistically similar by race-ethnicity, age, gender, primary language, payer type, and residence type. According to their EHR, approximately 70% of patients invited to participate identified as being of Hispanic ethnicity or as Black or African American, Asian, or another non-White race (Table 1). 16% of the population had Medicaid coverage and approximately 9% resided in rural areas.

**TABLE 1:** Demographic characteristics of study populations.

Characteristic	Overall^a^ N = 20,400	Digital-only^a^ N = 10,202	Brochure plus digital^a^ N = 10,198	*p*-value^b^
Age Group				0.13
18–35	5,073 (24.9%)	2,522 (24.7%)	2,551 (25.0%)	
36–45	3,817 (18.7%)	1,846 (18.1%)	1,971 (19.3%)	
46–55	3,909 (19.2%)	1,999 (19.6%)	1,910 (18.7%)	
56–65	3,761 (18.4%)	1,906 (18.7%)	1,855 (18.2%)	
66+	3,840 (18.8%)	1,929 (18.9%)	1,911 (18.7%)	
Gender				0.4
Female	13,348 (65.4%)	6,707 (65.7%)	6,641 (65.1%)	
Male	7,048 (34.5%)	3,494 (34.2%)	3,554 (34.8%)	
Another Gender Identity	4 (0.0%)	1 (0.0%)	3 (0.0%)	
Race-Ethnicity				0.9
Another Race^c^	459 (2.2%)	236 (2.3%)	223 (2.2%)	
Asian	4,656 (22.8%)	2,327 (22.8%)	2,329 (22.8%)	
Black or African American	3,655 (17.9%)	1,834 (18.0%)	1,821 (17.9%)	
Hispanic	5,644 (27.7%)	2,811 (27.6%)	2,833 (27.8%)	
Unknown	328 (1.6%)	174 (1.7%)	154 (1.5%)	
White or Caucasian	5,658 (27.7%)	2,820 (27.6%)	2,838 (27.8%)	
Payer Type				0.8
Medicaid	3,283 (16.1%)	1,634 (16.0%)	1,649 (16.2%)	
Other Insurance	17,117 (83.9%)	8,568 (84.0%)	8,549 (83.8%)	
Primary Language				0.5
English	19,096 (93.6%)	9,563 (93.7%)	9,533 (93.5%)	
Spanish	1,304 (6.4%)	639 (6.3%)	665 (6.5%)	
Residence Type				0.6
Rural	1,813 (8.9%)	916 (9.0%)	897 (8.8%)	
Urban	18,587 (91.1%)	9,286 (91.0%)	9,301 (91.2%)	

^a^ n (%); Percentages are based on characteristic total by column.

^b^ Pearson’s Chi-squared test; Fisher’s exact test.

^c^ Another Race includes American Indian or Alaska Native, Native Hawaiian or Pacific Islander, Other, and Two or More Races.

### 3.2 Enrollment

1,532 individuals enrolled in Geno4ME (7.5% of eligible patients); 54.9% of those individuals were identified in their EHR as being Hispanic, Black or African American, Asian, or another non-White race (Table 2). Enrollment rates for the *Brochure Plus Digital* and *Digital-Only* outreach methods were 7.8% and 7.3%, respectively. Enrollment rates for some subgroups were higher than average, including individuals insured through Medicaid (8.7%), individuals living in zip codes classified as rural (10.6%), and individuals identified in their EHR as White or Caucasian (12.2%). Enrollment was lower than average among individuals whose primary language was Spanish (2.8%), individuals identified in their EHR as Hispanic (5.4%), and individuals identified in their EHR as Black or African American (5.6%).

**TABLE 2:** Enrollment rates by outreach approach.

	Overall N = 20,400	Digital-only N = 10,202	Brochure plus digital N = 10,198
Overall Enrolled	1,532 (7.5%)^a^	745 (7.3%)^1^	787 (7.8%)^1^
Age Group			
18–35	294 (5.8%)	147 (5.8%)	147 (5.8%)
36–45	289 (7.6%)	132 (7.2%)	157 (8.1%)
46–55	320 (8.2%)	162 (8.1%)	158 (8.3%)
56–65	305 (8.1%)	141 (7.4%)	164 (8.9%)
66+	324 (8.5%)	163 (8.5%)	161 (8.5%)
Gender			
Female	1,064 (8.0%)	513 (7.7%)	551 (8.4%)
Male	468 (6.7%)	232 (6.6%)	236 (6.7%)
Another Gender Identity	0 (0.0%)	0 (0.0%)	0 (0.0%)
Race-Ethnicity			
Another Race^b^	33 (7.2%)	12 (5.1%)	21 (9.5%)
Asian	301 (6.5%)	134 (5.8%)	167 (7.2%)
Black or African American	204 (5.6%)	101 (5.5%)	103 (5.7%)
Hispanic	303 (5.4%)	151 (5.4%)	152 (5.4%)
Unknown	9 (2.8%)	3 (1.7%)	6 (4.0%)
White or Caucasian	682 (12.2%)	344 (12.2%)	338 (12.1%)
Payer Type			
Medicaid	284 (8.7%)	132 (8.1%)	152 (9.3%)
Other Insurance	1,248 (7.3%)	613 (7.2%)	635 (7.5%)
Primary Language			
English	1,496 (7.9%)	729 (7.6%)	767 (8.1%)
Spanish	36 (2.8%)	16 (2.5%)	20 (3.0%)
Residence Type			
Rural	190 (10.6%)	84 (9.2%)	106 (12.0%)
Urban	1,342 (7.3%)	661 (7.1%)	681 (7.4%)

^a^ Enrolled n (%).

^b^ Another race includes American Indian or Alaska Native, Native Hawaiian or Pacific Islander, Other, and Two or More Races.

In the overall population, there was no significant difference in enrollment rate between the *Brochure Plus Digital* and *Digital-Only* outreach methods (Table 3). We divided our study population by the demographic characteristics used to create our stratified sample (race-ethnicity, payer type, and residence type), and examined whether the *Brochure Plus Digital* outreach approach was associated with significantly higher enrollment for any subgroups. The adjusted rate of enrollment was 1.25 times greater for Asian patients in the *Brochure Plus Digital* approach than for Asian patients in the *Digital-Only* group, significant after adjusting for age, gender, payer, and residence type (*p* = 0.047). Additionally, the adjusted rate of enrollment was greater for patients who resided in rural areas using the *Brochure Plus Digital* approach compared to the *Digital-Only* approach (adjusted IRR = 1.29, *p* = 0.061). Adjusted enrollment rates did not differ significantly between the two outreach approaches for other demographic stratifications tested. Unadjusted rates were comparable to the adjusted rates. We also examined differences in effectiveness of outreach method by gender and age and observed no significant effect (data not shown). Finally, although not a key outcome in this article, it is worth noting that among the enrolled population, completion and return of the at-home DNA test kit was comparable across both outreach groups (∼70% DNA kit return rate).

**TABLE 3:** Strength of association between outreach approach and enrollment.

Population	N	Adjusted IRR^a^ (95% CI)	Adjusted *p*-value
Full Study	20,400	1.07 (0.97, 1.18)	0.19
*Subgroups*			
*by Race-Ethnicity* ^b^			
Another Race^c^	459	1.76 (0.89, 3.48)	0.11
Asian	4,656	1.25 (1, 1.56)	0.047
Black or African American	3,655	1.04 (0.79, 1.35)	0.79
Hispanic	5,644	1.00 (0.8, 1.24)	>0.9
Unknown	328	2.05 (0.58, 7.27)	0.26
White or Caucasian	5,658	0.99 (0.86, 1.14)	>0.9
*by Payer Type* ^d^			
Medicaid	3,283	1.13 (0.91, 1.4)	0.13
Other Insurance	17,117	1.05 (0.95, 1.17)	0.35
*by Residence Type* ^e^			
Rural	1,813	1.29 (0.99, 1.69)	0.061
Urban	18,587	1.04 (0.94, 1.15)	0.46

^a^ Incidence Rate Ratio (IRR): Rate of enrollment for the Brochure Plus Digital group divided by rate of enrollment via Digital-Only group.

^b^ Adjusted for Age, Gender, Payer, and Residence Types.

^d^ Adjusted for Age, Gender, Race-Ethnicity, and Residence Types.

^e^ Adjusted for Age, Gender, Race-Ethnicity, and Payer Types.

^c^ Another race includes American Indian or Alaska Native, Native Hawaiian or Pacific Islander, Other, and Two or More Races.

### 3.3 Mode of enrollment

**FIGURE 2:**
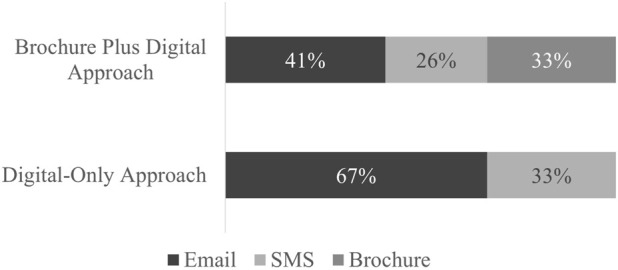
Link used for enrollment by outreach approach.

The type of link (brochure QR code, email link, or SMS link) that patients used to enroll in the study was tracked (Figure 2). Enrollment via email link was favored among both outreach approaches (67% for *Digital-Only* and 41% for *Brochure Plus Digital*). A third of enrollees in the *Brochure Plus Digital* group used the QR code printed on the mailed brochure to enroll. Frequency distribution of the links used for enrollment by demographic group is included in the Appendix (Table 1). Because type and number of contacts made were affected by outreach group and individual response (e.g., if someone received a mailed brochure and enrolled immediately, they would not have received a follow-up text or email), statistical comparisons were not made between outreach approaches.

## 4 Discussion

In this study, we used disproportionate stratified sampling to recruit a diverse patient population to participate in Geno4ME, a WGS program. We used different combinations of mail-based paper brochure, email, and SMS outreach to create two outreach approaches: *Brochure Plus Digital* and *Digital-Only*. The two approaches performed similarly, and 7.5% of all eligible patients enrolled in the program. Across both approaches, links provided via email were most used for enrollment. Over 50% of the enrolled population identified in their EHR as being Hispanic, Black of African American, Asian, or another non-White race. While the enrolled population was diverse, enrollment rates were lower than average for patients whose primary language was Spanish, patients identified in their EHR as Hispanic, and patients identified in their EHR as Black or African American. Enrollment rates were higher than average for patients insured through Medicaid, those living in rural areas, and who identified in their EHR as White or Caucasian. Subgroup analysis showed limited significant differences in effectiveness for the two outreach approaches by demographics.

7.8% of the *Brochure Plus Digital* group and 7.3% of the *Digital-Only* group enrolled in Geno4ME. Interestingly, patients with Medicaid coverage (representing lower socioeconomic status) and those in rural areas enrolled at higher rates indicating that there is interest and engagement from these underrepresented groups when the option to participate in genetic testing is presented. Contextualizing these rates is challenging due to the limited number of studies that have explored population outreach for genetic testing and the differences in sampling, recruitment methods, and study populations between Geno4ME and the studies that do exist. One such study is the BabySeq Project, which explored parents’ interest in newborn genomic sequencing and had an overall enrollment rate of 6.9% using a two-step enrollment process (Genetti et al., 2019). While 10% of all families approached agreed to attend an enrollment session with a genetic counselor, 67% of attendees then enrolled in the program. The authors note that the study population was largely non-Hispanic White and their findings may not be generalizable to more diverse populations, suggesting this as an area of exploration for future studies. Relatedly, Geisinger Health System’s MyCode biobanking program had a consent rate of more than 85% when eligible patients met with clinic staff or researchers who explained the program during outpatient visits (Carey et al., 2016). Carey et al. note that over 95% of the regional population served by the health system is White, limiting opportunities to study disparities among racial and ethnic groups or differences in genetic variant frequencies. Both studies highlight the effectiveness of direct engagement with patients to encourage their enrollment in WGS programs, which is a strategy that Geno4ME was unable to employ at its scale. Both studies also underscore current gaps in outreach and engagement with patients of color and other populations traditionally underrepresented in genomics.

There are many potential reasons why enrollment in genetic testing did not happen at a higher rate. For example, it could be due to limited awareness of genetic testing’s potential utility among the public; according to one study, only half of U.S. survey respondents were aware that testing could be used to screen for inherited cancer risks (Roberts et al., 2022). Other studies have also demonstrated a need for more information as a reason to decline testing (Neghina and Anghel, 2010). A systematic review of studies on genetic screening in the general healthy population surfaced several barriers to screening including psychosocial factors (such as anxiety, fear, and worry about screening), potential negative psychological impacts, mistrust, disinterest, possibility of receiving unwanted information, belief that you are low risk, and moral or ethical reasons (Shen et al., 2022). An additional reason cited was dislike of blood (Neghina and Anghel, 2010; Shen et al., 2022), but this seemed less relevant for this study as the screening used saliva; however, it should be a consideration for programs that require a blood draw for screening.

Despite more than 50% of enrolled Geno4ME patients being identified in their EHR as Hispanic, Black or African American, Asian, or another non-White race, patients from these demographic groups enrolled at lower rates than the overall population. Previous studies have highlighted concerns expressed by patients of color about participating in genomics due to mistrust rising from extensive past harms and structural barriers stemming from systemic racism. In one qualitative study with Black patients about genetic study participation, a third of interviewees discussed past and current racial discrimination in research as a frame through which they thought about participating (Kikut et al., 2022). Another study explored Black men’s attitudes towards genomics through focus groups and found that barriers to participation included lack of terminology understanding, health system mistrust, reluctance to seek medical care, and unfavorable attitudes towards medical research (Rogers et al., 2018). Surveys have also found that concerns about test result privacy, health insurance discrimination, and life insurance discrimination were significantly higher among patients of color (Lemke et al., 2021). Taken together, our findings and these studies emphasize the importance of investing in intentional relationship building with patients of color and other populations traditionally underrepresented in genomics to address these concerns and ensure that the potential benefits of genomics are accessible to all patients.

Outreach was equally effective across our two approaches (7.8% of patients in the *Brochure Plus Digital* group enrolled in Geno4ME compared to 7.3% in the *Digital-Only* group), and the inclusion of a mail-based paper brochure did not have a significant impact on general outreach effectiveness. While enrollment rates did not differ significantly between the two outreach approaches for most demographic groups, Asian patients and patients residing in rural areas both enrolled at higher rates when receiving the mail-based brochure (adjusted IRR = 1.25, *p* = 0.047 and adjusted IRR = 1.29, *p* = 0.061, respectively). The reasons for these differences in enrollment rates are unclear and warrant future research. Of note, the cost per enrollment was substantially higher when paper brochures were included (due to processing and material costs associated with printing and mailing); but, depending on the patient populations that future studies hope to engage, mail-based outreach could be an important part of multi-modal WGS recruitment strategies.

While this study contributes to our understanding of how to effectively recruit a diverse patient population to participate in WGS programs, it also creates opportunities for future research to build upon. As noted earlier, it is unclear why Asian patients and patients residing in rural areas enrolled at higher rates when receiving the brochure, and unique considerations for these and other populations should be explored. Additionally, patients whose primary language was Spanish enrolled in Geno4ME at notably lower rates than average regardless of outreach approach, and future research should examine the unique barriers to participating in WGS initiatives faced by this population (even when all materials are offered in Spanish). Future studies could also focus on post-enrollment participation by examining rates of DNA kit return and the extent to which these rates vary across demographic groups, as well as whether and how patients engage with their primary care provider or genetic counselor to discuss their results and any variation by demographic groups. Future studies could also explore patient experiences with participation and the extent to which they felt their test results were actionable and improved their sense of wellbeing. Finally, an opportunity exists to engage with patients who specifically opted not to participate in WGS programs, rather than just not respond, to better understand barriers and concerns related to genomics participation.

This study has several important limitations. First, Geno4ME consent and enrollment was available only through a link or QR code, thus requiring all patients to have some degree of access to technology, which may have limited enrollment or minimized differences between brochure and digital outreach approaches. Additionally, email and SMS outreach were combined into a broader digital outreach category in our study, which may hide variations in effectiveness for these different methods generally or for specific demographic groups. Furthermore, our study population and outreach analysis relied on EHR data for patient demographic information, which might not have been collected in a standardized way or reflect how patients choose to identify. Finally, we oversampled from groups that were well-represented in our eligible pool, and therefore we had limited enrollment from racial identities that were not oversampled (American Indian or Alaska Native, Native Hawaiian or Pacific Islander, Other, and Two or More Races). We combined these small enrollment groups into “Another Race” for purposes of our analysis, which combines diverse populations into one group and may minimize differences in outreach effectiveness and preferences between different identities.

Given the anticipated role of genomics in medicine, understanding the most effective approaches to patient engagement will be vital for the future of healthcare. Further recognizing how outreach preferences vary across demographic groups and the extent to which certain groups face unique barriers to participating in genomics will be critical for engaging populations traditionally underrepresented in genomics and addressing health disparities. Digital outreach strategies could be used independently for cost-effective enrollment in population-level WGS programs or in combination with strategies to build relationships with patients, particularly with patients traditionally underrepresented in genomics. This initial outreach is the first step in ensuring that genomics helps create a future with greater opportunities for health for all patients.

## Data Availability

The raw data supporting the conclusion of this article will be made available by the authors, without undue reservation.
