# Supplementary material for: Participation in genetic screening: testing different outreach methods across a diverse hospital system based patient population
Source: Front Genet. 2023 Oct 12;14:1272931. doi: 10.3389/fgene.2023.1272931 (PMC10602775; doi:10.3389/fgene.2023.1272931)
Supplement: Supplementary file 1 [file Table1.docx]

**Supplementary Material**

Supplemental Table 1. Link Used for Enrollment by Outreach Approach

|  | Digital-Only | | Brochure Plus Digital | | |
| --- | --- | --- | --- | --- | --- |
| Characteristic | Email 495 (67%)^1^ | SMS 248 (33%)^1^ | Brochure 259 (33%)^1^ | Email  (41%)^1^ | SMS 203 (26%)^1^ |
| Age Group |  |  |  |  |  |
| 18-35 | 94 (63.9%) | 53 (36.1%) | 50 (34.0%) | 62 (42.2%) | 35 (23.8%) |
| 36-45 | 94 (71.2%) | 38 (28.8%) | 54 (34.4%) | 71 (45.2%) | 32 (20.4%) |
| 46-55 | 106 (65.4%) | 56 (34.6%) | 58 (36.7%) | 52 (32.9%) | 48 (30.4%) |
| 56-65 | 88 (62.9%) | 52 (37.1%) | 51 (31.1%) | 66 (40.2%) | 47 (28.7%) |
| 66+ | 113 (69.8%) | 49 (30.2%) | 46 (28.7%) | 73 (45.6%) | 41 (25.6%) |
| Gender |  |  |  |  |  |
| Female | 333 (65.0%) | 179 (35.0%) | 167 (30.4%) | 238 (43.3%) | 145 (26.4%) |
| Male | 162 (70.1%) | 69 (29.9%) | 92 (39.0%) | 86 (36.4%) | 58 (24.6%) |
| Race-Ethnicity |  |  |  |  |  |
| Another Race^2^ | 5 (41.7%) | 7 (58.3%) | 8 (38.1%) | 9 (42.9%) | 4 (19.0%) |
| Asian | 98 (73.1%) | 36 (26.9%) | 55 (32.9%) | 76 (45.5%) | 36 (21.6%) |
| Black or African American | 63 (62.4%) | 38 (37.6%) | 31 (30.4%) | 39 (38.2%) | 32 (31.4%) |
| Hispanic | 92 (60.9%) | 59 (39.1%) | 49 (32.2%) | 62 (40.8%) | 41 (27.0%) |
| Unknown | 2 (66.7%) | 1 (33.3%) | 3 (50.0%) | 1 (16.7%) | 2 (33.3%) |
| White or Caucasian | 235 (68.7%) | 107 (31.3%) | 113 (33.4%) | 137 (40.5%) | 88 (26.0%) |
| Payer Type |  |  |  |  |  |
| Medicaid | 83 (62.9%) | 49 (37.1%) | 47 (30.9%) | 57 (37.5%) | 48 (31.6%) |
| Other Insurance | 412 (67.4%) | 199 (32.6%) | 212 (33.4%) | 267 (42.1%) | 155 (24.4%) |
| Primary Language |  |  |  |  |  |
| English | 483 (66.4%) | 244 (33.6%) | 254 (33.2%) | 316 (41.3%) | 196 (25.6%) |
| Spanish | 12 (75.0%) | 4 (25.0%) | 5 (25.0%) | 8 (40.0%) | 7 (35.0%) |
| Residence Classification |  |  |  |  |  |
| Rural | 54 (65.1%) | 29 (34.9%) | 40 (37.7%) | 41 (38.7%) | 25 (23.6%) |
| Urban | 441 (66.8%) | 219 (33.2%) | 219 (32.2%) | 283 (41.6%) | 178 (26.2%) |
| ^1^n (%); Percentages are based on characteristic level subtotal per Outreach Approach ^2^ Another race includes *American Indian or Alaska Native, Native Hawaiian or Pacific Islander, Other,* and *Two or More Races* | | | | | |
